# How much of the variance in functional outcome related to intracerebral hemorrhage volume is already apparent in neurological status at admission?

**DOI:** 10.1007/s00415-024-12427-9

**Published:** 2024-05-22

**Authors:** Vincent Geest, Janja Pretnar Oblak, Katarina Šurlan Popović, Jawed Nawabi, Sarah Elsayed, Constanze Friedrich, Maik Böhmer, Burak Akkurt, Peter Sporns, Andrea Morotti, Frieder Schlunk, Paul Steffen, Gabriel Broocks, Lukas Meyer, Uta Hanning, Götz Thomalla, Susanne Gellissen, Jens Fiehler, Senta Frol, Helge Kniep

**Affiliations:** 1 Department of Neuroradiology, University Medical Center Hamburg-Eppendorf, Hamburg, Germany; 2 Department of Neurology, University Medical Center Hamburg-Eppendorf, Hamburg, Germany; 3 Department of Neurology, University Medical Center Ljubljana, Ljubljana, Slovenia; 4 Medical Faculty, University of Ljubljana, Ljubljana, Slovenia; 5 Department of Neuroradiology, University Medical Center Ljubljana, Ljubljana, Slovenia; 6 Department of Radiology, University Hospital Muenster, Muenster, Germany; 7 Department of Neuroradiology, Charité - Universitätsmedizin Berlin, Berlin, Germany; 8 Department of Neuroradiology, Clinic for Radiology and Nuclear Medicine, University Hospital Basel, Basel, Switzerland; 9 Department of Clinical and Experimental Sciences, Neurology Unit, University of Brescia, Brescia, Italy

**Keywords:** Intracerebral hemorrhage, Stroke, Mediation analysis

## Abstract

**Background:**

Hematoma volume is a major pathophysiological hallmark of acute intracerebral hemorrhage (ICH). We investigated how the variance in functional outcome induced by the ICH volume is explained by neurological deficits at admission using a mediation model.

**Methods:**

Patients with acute ICH treated in three tertiary stroke centers between January 2010 and April 2019 were retrospectively analyzed. Mediation analysis was performed to investigate the effect of ICH volume (0.8 ml (5% quantile) versus 130.6 ml (95% quantile)) on the risk of unfavorable functional outcome at discharge defined as modified Rankin Score (mRS) ≥ 3 with mediation through National Institutes of Health Stroke Scale (NIHSS) at admission. Multivariable regression was conducted to identify factors related to neurological improvement and deterioration.

**Results:**

Three hundred thirty-eight patients were analyzed. One hundred twenty-one patients (36%) achieved mRS ≤ 3 at discharge. Mediation analysis showed that NIHSS on admission explained 30% [13%; 58%] of the ICH volume-induced variance in functional outcome at smaller ICH volume levels, and 14% [4%; 46%] at larger ICH volume levels. Higher ICH volume at admission and brainstem or intraventricular location of ICH were associated with neurological deterioration, while younger age, normotension, lower ICH volumes, and lobar location of ICH were predictors for neurological improvement.

**Conclusion:**

NIHSS at admission reflects 14% of the functional outcome at discharge for larger hematoma volumes and 30% for smaller hematoma volumes. These results underscore the importance of effects not reflected in NIHSS admission for the outcome of ICH patients such as secondary brain injury and early rehabilitation.

## Introduction

Hematoma volume is a major pathophysiological hallmark of acute primary intracerebral hemorrhages (ICH) and a significant predictor of ICH patients’ outcome [4, 22]. However, while preliminary results of the ongoing ENRICH trial (NCT02880878) investigating minimal invasive parafascicular ICH evacuation are encouraging, randomized controlled trials so far have failed to show a substantial benefit of acute hematoma volume reduction for functional outcome [12, 27]. The supposed contradiction demonstrates that the association between ICH volume at admission and functional outcome is not yet fully understood and raises the question when and to what extent the effect of ICH volume reduction on functional outcome becomes clinically apparent.

Several studies describe early neurological status at admission quantified by the National Institutes of Health Stroke Scale (NIHSS) as a reliable predictor of functional outcome in ICH patients [8, 25, 29]. These results indicate that a substantial proportion of the effect of ICH volume on functional outcome is explained by the extent of neurological deficits on admission reflecting the early primary brain injury by ICH. This hypothesis is challenged by studies that point out the larger impact of clinical shifts after admission for the outcome of ICH patients, like secondary neurological deteriorations due to hematoma expansion [20, 23, 37].

We addressed this question by analyzing what proportion of the ICH volume-induced variance in functional outcome at discharge is explained by neurological deficits at admission and what proportion in functional outcome is explained by effects not reflected in the early neurological status using a mediation model. NIHSS as established parameter to quantify the extent of focal neurological deficits on admission [10, 31, 35] was chosen as mediator over the also well-established ICH score as the latter takes into account other factors as clinical status and does not predict functional outcome beyond mortality. Our hypothesis was that admission NIHSS explained more of the outcome variance in smaller compared to larger volume ICHs [11, 32].

## Methods

### Study design

This multicenter retrospective study is based on pooled individual data of patients with spontaneous ICH, treated in three high-volume tertiary stroke centers (University Medical Centers of Hamburg-Eppendorf, Münster and Ljubljana) between May 2009 and November 2020. Inclusion criteria were: (1) age ≥ 18 years; (2) spontaneous ICH confirmed on non-enhanced computed tomography (NECT) or NECT and computed tomography angiography (CTA) performed routinely on admission; (3) availability of relevant clinical data points. Patients were excluded if they had a secondary ICH from head trauma, hemorrhagic transformation of ischemic infarction, brain tumor, cerebral aneurysm or vascular malformation. Baseline patient characteristics were retrieved from medical records, including NIHSS at admission and modified Rankin Score (mRS) at discharge. In addition, vascular risk factors (hypertension, diabetes mellitus) and follow-up procedures, such as craniectomy or intraventricular drainage placement, were obtained from clinical records and follow-up NECT. Good functional outcome was defined as mRS ≤ 3 at discharge. The study was approved by the ethics committees of the participating centers (Ethik-Kommission der Ärztekammer Hamburg, Ethik-Komission der Charité Berlin, commission of the Republic of Slovenia for medical ethics) and written informed consent was waived by the institutional review boards. All study protocols and procedures were conducted in accordance with the Declaration of Helsinki. The Strengthening the Reporting of Observational Studies in Epidemiology (STROBE) guideline was used for reporting this observational study [34]. The deidentified data are available from the corresponding author upon reasonable request.

### Image acquisition

NECT scans were performed using standard clinical parameters with axial < 5 mm section thickness. All datasets were inspected for quality and excluded in case of severe motion artifacts. In detail, the images were acquired on the following scanners:

256 slice scanner (Philips iCT 256) with 120 kV, 280–320 mA, < 5.0 mm slice reconstruction and < 0.5 mm in-plane resolution and CTA with 100−120 kV, 260–300 mA, 1.0 mm slice reconstruction, 5-mm MIP reconstruction with 1 mm increment, 0.6-mm collimation, 0.8 pitch, H20f soft kernel, 80 mL highly iodinated contrast medium and 50 mL 0.9% sodium chloride solution flush at 4 mL/s; scan starts 6 s after bolus tracking at the level of the ascending aorta.

128 slice scanner (SOMATOM Definition Flash) with 120 kV, 280 mA, < 5.0 mm slice reconstruction and < 0.5 mm in-plane resolution and CTA with 100–120 kV, between 260 and 300 mA, 1.0-mm slice reconstruction, 5-mm MIP reconstruction with 1 mm increment, 0.5-mm collimation, 0.8 pitch, H20f soft kernel, 60 mL highly iodinated contrast medium and 30 mL 0.9% sodium chloride solution flush at 4 mL/s; scan starts 6 s after bolus tracking at the level of the ascending aorta.

80 slice scanner (Toshiba Aquilion Prime) with 120 kV, 280 mA, < 5.0 mm slice reconstruction and < 0.5 mm in-plane resolution and CTA with 100–120 kV, 260–300 mA, 1.0 mm slice reconstruction, 5-mm MIP reconstruction with 1 mm increment, 0.5-mm collimation, 0.8 pitch, H20f soft kernel, 60 mL highly iodinated contrast medium and 30 mL 0.9% sodium chloride solution flush at 4 mL/s; scan starts 6 s after bolus tracking at the level of the ascending aorta.

40 slice scanner (Siemens SOMATOM Sensation OPEN) with 120 kV, 280 mA, 3.0 mm slice reconstruction, 0.6-mm in-plane resolution and collimation 20 × 0.6 mm, CTA with 120 kV, 160 mA, CAREdose, collimation 40 × 0.6 mm, 0.75-mm slice reconstruction, 3 mm MIP reconstruction with 1 mm increment, 1.2 pitch, B20f smooth, 80 ml highly iodinated contrast medium, 40 ml 0.9% sodium chloride solution, flow 5 ml/s, star scan 3 s after bolus tracking at the level of the ascending aorta.

### Image analysis

NECT scans were analyzed by three experienced neuroradiologists (JN, SE, VG) for the following ICH features: (1) ICH volume; (2) ICH location; (3) intraventricular hemorrhage; (3) craniectomy in the follow-up NECT scans. ICH locations were differentiated as basal ganglia, thalamus, lobe, brain stem, pons, and cerebellum. ICH volumes were segmented semi-automatically based on admission NECT images. Regions of interest (ROIs) were delineated using Analyze 11.0 Software (Biomedical Imaging Resource, Mayo Clinic, Rochester, MN). Discrepancies were settled by joint discussion.

### Statistical analysis

Standard descriptive statistics were used for all study end points. Univariable distribution of metric variables was described with mean and interquartile range and categorical variables with absolute and relative frequencies.

Multivariable logistic regression analysis was performed to identify the independent predictors of good functional outcome defined as mRS ≤ 3 at discharge. Multivariable linear regression was used to identify the independent predictors of neurological status defined as NIHSS on admission.

**Fig. 1:**
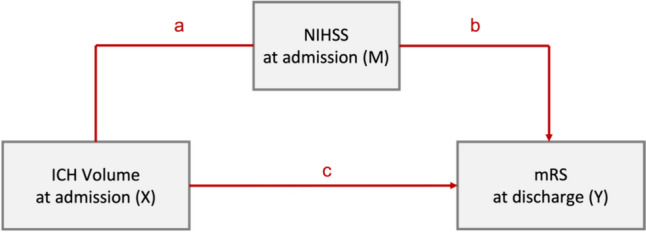
Mediation model design

Mediation analysis [3, 18] was used to evaluate to what extent neurological status at admission explains functional outcome at discharge for smaller ICHs compared larger ICHs (Fig. 1). Based on the 5% and 95% quantiles of our study population, smaller hematoma levels were defined as 0.8 ml whereas larger hematoma levels were defined as 130.6 ml. Mediation models consider the impact of a mediator variable that is hypothesized to transmit the influence of independent variables onto an outcome. The underlying regression models were defined according to Mackinnon and Dwyer [24]. Mediation analysis was conducted in adherence to the requirements defined by Baron and Kenny [3], employing algorithms proposed by Imai and Keele [15, 16] that allow estimation of causal mediation effects for linear and nonlinear relationships with continuous and discrete mediators, and various types of outcome variables. Confidence intervals of the mediation analysis were derived using quasi-Bayesian approximation.

Baseline factors that independently predict unexpected neurological deterioration or improvement after admission and thereby reducing the explanatory power of the neurological status at admission were identified using multivariable logistic regression analysis. Unexpected neurological deterioration was defined as shift from promising early neurological status with NIHSS ≤ 6 to unfavorable functional outcome with mRS > 3, unexpected neurological improvement was defined vice versa.

For all regression models, relevant factors were determined using backward variable selection procedures based on the Akaike information criterion. Adjusted odds ratios (aOR) and adjusted regression coefficients (aCO) with 95% confidence intervals (CI) and p values were calculated for selected variables. Two-sided p value < 0.05 was considered to be statistically significant. All analysis were carried out using R 3.6.2 with the *mediation* package 4.5.0 [36].

## Results

**Fig. 2:**
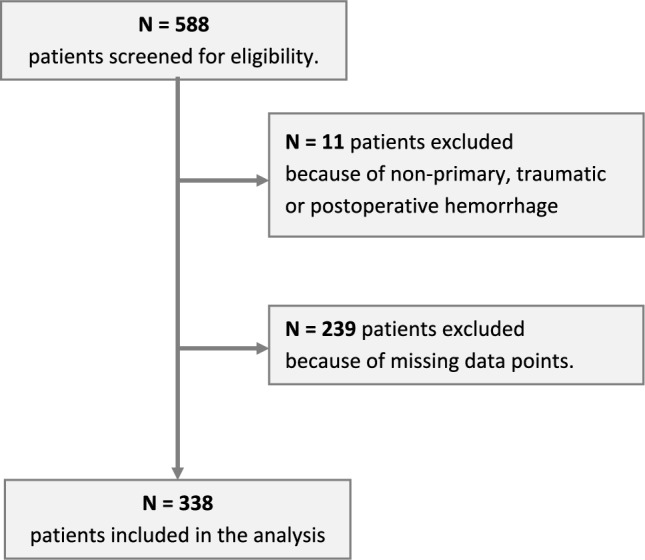
Patient inclusion flow chart

In total, 338 patients fulfilled the inclusion criteria and were analyzed (Fig. 2). Mean age was 71.3 years (standard deviation (SD) 13.7), 46% were female. Mean NIHSS at admission was 8 (interquartile range (IQR) 1–15) and mean ICH volume was 35.9 ml (SD 46.4). One hundred twenty-one patients (36%) achieved good outcome with mRS ≤ 3 at discharge. Compared to those with poor outcome (mRS ≥ 4 at discharge), these patients had significantly smaller hemorrhage volumes with a lower proportion of intraventricular hemorrhages and a higher proportion of lobar hemorrhages at admission. Correspondingly, their admission NIHSS was lower and their admission GCS was higher. The proportion of craniotomies was significantly higher in patients with poor outcome (Table 1).

**Table 1:** Study cohorts’ characteristics dichotomized by clinical outcome

	Total	mRS 0–3 at discharge	mRS 4–6 at discharge	*p* value
	*N* = 338	*N* = 121	*N* = 217	
Age (years), mean (SD)	71.3 (± 13.7)	69.6 (± 14.4)	72.3 (± 13.3)	0.122^1^
Female sex, n (%)	156 (46%)	55 (45%)	101 (47%)	0.847^2^
Hypertension, n (%)	274 (81%)	92 (76%)	182 (84%)	0.078^2^
Diabetes, n (%)	57 (17%)	20 (17%)	37 (17%)	0.902^2^
Antiplatelet treatment, n (%)	76 (22%)	26 (21%)	50 (23%)	0.743^2^
Anticoagulation treatment, n (%)	100 (30%)	35 (29%)	65 (30%)	0.843^2^
Admission				
NIHSS, median (IQR)	8 (1–15)	3 (1–8)	12 (2–17)	0.001^1^
GCS, median (IQR)	14 (11–15)	15 (13–15)	12 (10–15)	< 0.001^1^
ICH volume at admission				< 0.001^1^
Mean (SD)	35.9 (± 46.4)	20.0 (± 26.7)	44.8 (± 52.4)	
Median (IQR)	19.3 (6.7–47.8)	9.4 (3.0–25.9)	24.4 (11.3–65.4)	
Min–Max	0.2–378.2	0.2–135.5	0.4–378.2	
Supratentorial ICH localization, n (%)	296 (88%)	107 (88%)	189 (87%)	0.722^2^
Lobar, n (%)	148 (44%)	62 (51%)	86 (40%)	0.039^2^
Basal ganglia, n (%)	128 (38%)	39 (32%)	89 (41%)	0.111^2^
Thalamus, n (%)	20 (6%)	6 (5%)	14 (6%)	0.577^2^
Infratentorial ICH localization, n (%)	42 (12%)	14 (12%)	28 (13%)	0.722^2^
Brainstem, n (%)	12 (4%)	2 (2%)	10 (5%)	0.159^2^
Cerebellum, n (%)	30 (9%)	12 (10%)	18 (8%)	0.615^2^
Intraventricular hemorrhage, n (%)	135 (40%)	24 (20%)	111 (51%)	< 0.001^2^
Clinical course				
Craniectomy, n (%)	49 (14%)	5 (4%)	44 (20%)	< 0.001^2^
mRS at discharge, median (IQR)	4 (3–5)	2 (1–3)	5 (4–6)	< 0.001^1^

Characteristics were compared between patients with mRS 0–3 and mRS 4–6 with the use of either Mann–Whitney U test^1^ for continuous variables and Chi-square test^2^ for categorical variables

*mRS* modified Rankin Scale, *ASPECTS* Alberta Stroke Program Early CT Score, *NIHSS* National Institutes Health Stroke Scale, *GCS* Glasgow Coma Scale, *ICH* intracerebral hemorrhage, *IQR* interquartile range

### Predictors of functional outcome

**Fig. 3:**
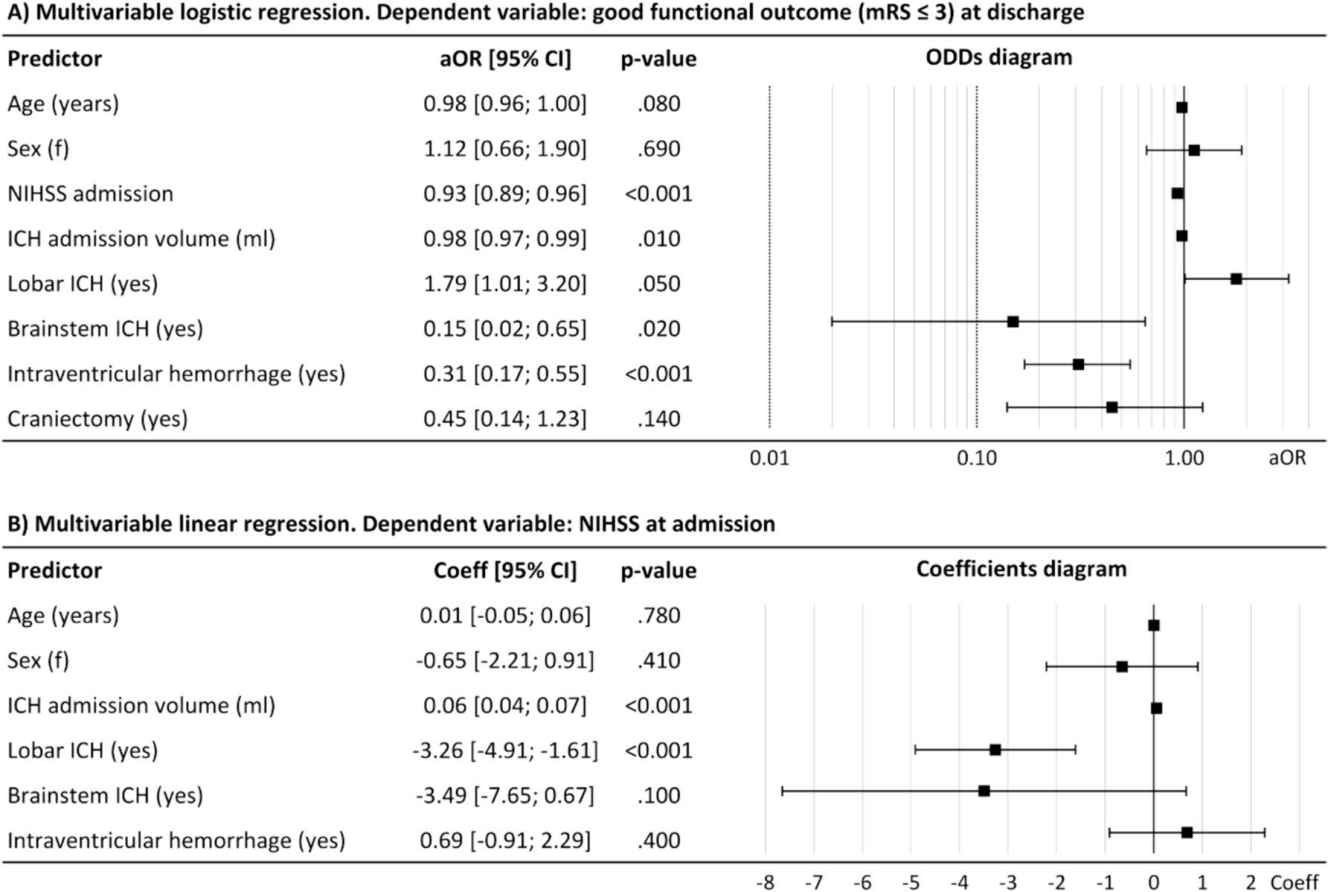
**A** Multivariable logistic regression. Dependent variable: good functional outcome (mRS ≤ 3) at discharge; **B** multivariable linear regression. Dependent variable: NIHSS at admission. *Coeff* coefficient, *CI* confidence interval, *ICH* intracerebral hemorrhage

In multivariable logistic regression analysis, probability of good functional outcome significantly decreased with higher admission NIHSS (odds ratio (OR) = 0.93; 95% CI [0.89;0.96]), higher admission ICH volume (0.98 [0.97; 0.99], bleeding location in brainstem (0.15 [0.02;0.65]), and intraventricular hemorrhage (0.31 [0.17; 0, 55]) (Fig. 3A).

### Predictors of NIHSS on admission

The mediation pathway with NIHSS at admission as dependent variable was analyzed using multivariable linear regression modeling as approximation for the ordinal NIHSS metric. NIHSS at admission was significantly positively associated with hematoma volume (coefficient = 0.06; 95% CI [0.04; 0.07]) and negatively associated with ICH location “lobar” (coefficient = −3.26, 95% CI [−4.91; −1.61]) (Fig. 3B).

### Mediation analysis

**Fig. 4:**
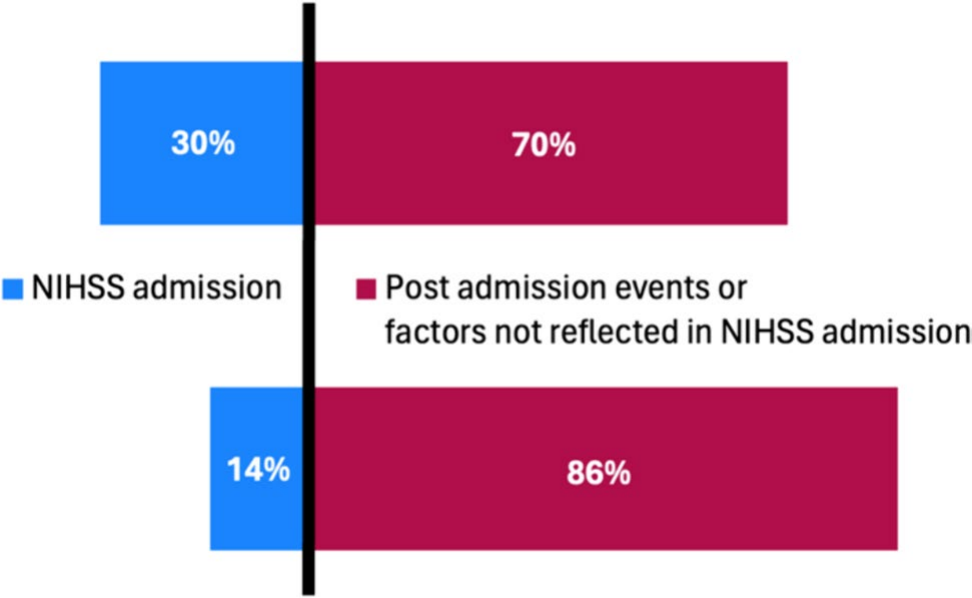
Mediated proportion of the effect of ICH volume on functional outcome at discharge

Mediation analysis showed a 36 [20; 49] percentage points (pp) decrease of probability of good functional outcome for patients with larger ICH volumes levels (defined as 130.6 ml) compared to patients with smaller ICH volume levels (defined as 0.8 ml). In patients with smaller ICH volume levels, NIHSS at admission explained 30% [13%; 58%] of the variance in functional outcome at discharge. In patients with larger ICH volume, NIHSS at admission explained 14% [3%; 55%] of the long-term functional outcome (Table 2, Fig. 4).

**Table 2:** Results of the mediation analysis

Predictor	Estimate	CI 2.5%	CI 97.5%	*p* value
Total effect of larger versus smaller ICH volume levels (increase of probability for mRS ≥ 4 at discharge)	36.24 pp	20 pp	49 pp	< 0.001
5% quantile of ICH volume				
Mediated effect explained by NIHSS admission	10.7 pp	5 pp	17 pp	< 0.001
Direct effect explained by post-admission events and other factors not reflected in NIHSS admission	30.9 pp	11 pp	45 pp	0.004
Proportion explained by NIHSS at admission	29.6%	13%	58%	< 0.001
95% quantile of ICH volume				
Mediated effect explained by NIHSS admission	5.4 pp	2 pp	11 pp	< 0.001
Direct effect explained by post-admission events and other factors not reflected in NIHSS admission	25.5 pp	11 pp	45 pp	0.004
Proportion explained by NIHSS at admission	13.8%	4%	46%	< 0.001

*NIHSS* National Institutes Health Stroke Scale, *CI* confidence interval, *ICH* intracerebral hemorrhage

### Predictors of unexpected neurological deterioration or improvement

**Fig. 5:**
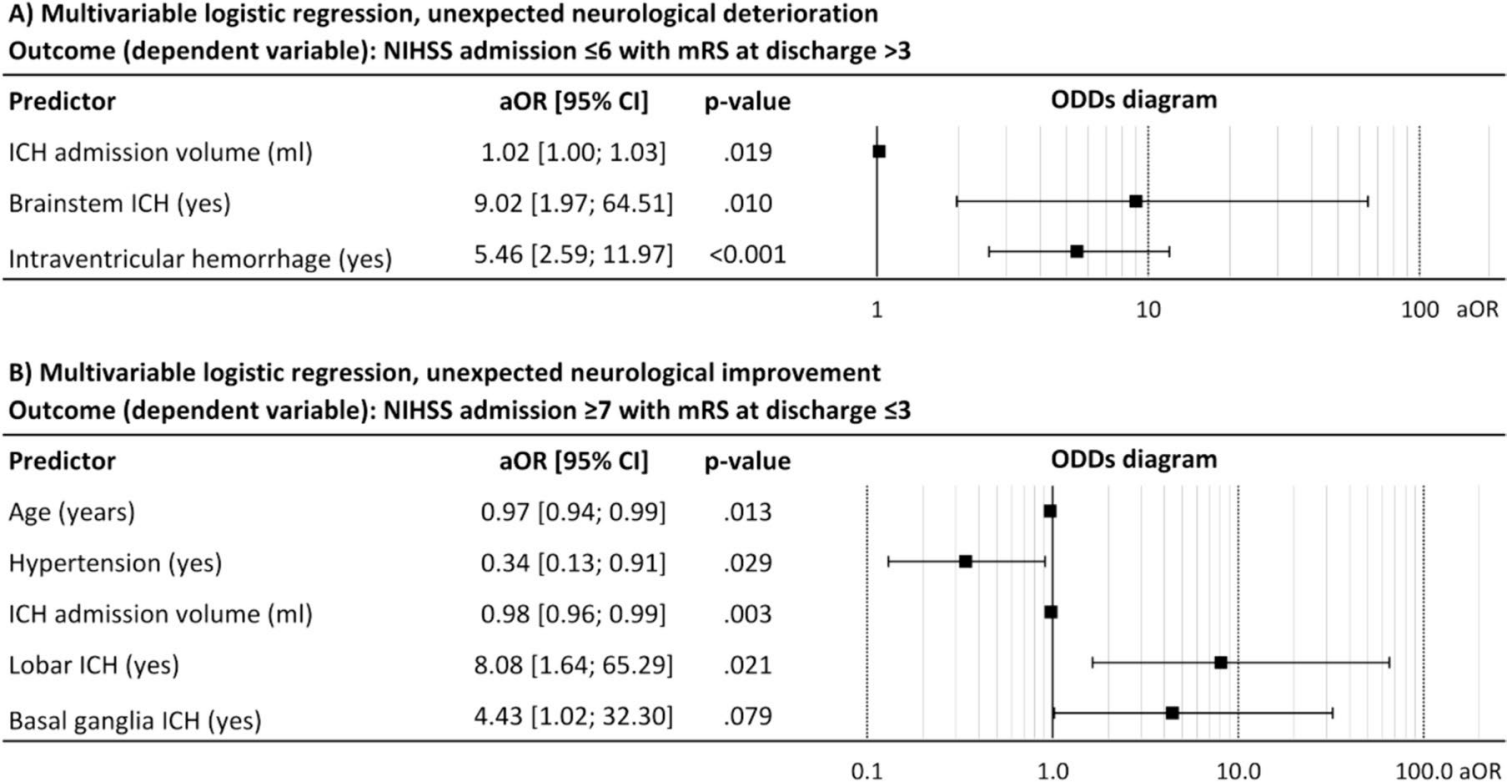
Predictors of unexpected neurological deterioration (**A** NIHSS admission ≤ 6 with mRS at discharge > 3) and improvement (**B** NIHSS admission ≥ 7 with mRS at discharge ≤ 3). *Coeff* coefficient, *CI* confidence interval, *ICH* intracerebral hemorrhage

The assessment of patient-specific factors increasing probability of unexpected neurological deterioration (defined as NIHSS admission ≤ 6 with mRS at discharge > 3) or improvement (NIHSS admission ≥ 7 with mRS at discharge ≤ 3) indicates that higher ICH volume at admission and brainstem or intraventricular location of ICH are relevant factors associated with neurological deterioration (Fig. 5A), while younger age, normotension, lower ICH volumes, and lobar location of ICH are predictors for unexpected neurological improvement (Fig. 5B).

## Discussion

Our analysis showed that the neurological status on admission as captured by NIHSS reflects 30% of the hematoma volume-induced variance in functional outcome at smaller levels of ICH volumes (defined as 5% quantile) and 14% of the variance in functional outcome at larger levels of ICH volumes (defined as 95% quantile). 70–86% of the effect of hematoma volume on clinical outcome are not reflected in NIHSS at admission. These results suggest that most of the brain injury caused by ICH manifests after admission and/or there are limitations of NIHSS in capturing the brain injury already present on admission.

Several studies have previously analyzed the association between hemorrhage volume, neurological status at admission, and functional outcome in patients with ICH. In concordance with our results, all have demonstrated that neurological assessment at admission using NIHSS is a predictor of functional outcome in ICH patients [5, 13, 14]. However, by formally testing NIHSS for the first time using a mediator analysis, we demonstrated that NIHSS at admission explained at most one-third of the effect of hemorrhage volume on functional outcome.

These results can be explained by studies that attribute great importance to the clinical course after admission for the outcome of ICH patients. In this phase, internal effects like hematoma expansion, perihematomal edema, ischemia and inflammation, blood related toxic effects, and hydrocephalus can cause secondary deterioration of ICH patients [20, 23, 37]. In addition, external post-admission effects associated with hemorrhage volume, such as the necessity and duration of intensive medical care (including side effects like respiratory infections), and effectiveness of rehabilitation likely contribute to the explanatory mismatch between admission NIHSS and functional outcome [6, 21, 33]. According to this hypothesis, the much lower explanatory power of the NIHSS for larger hematoma volumes (14%) compared to smaller volumes (30%) indicates a growing impact of these internal and external effects on the outcome with increasing hematoma volume. Previous studies already reported that patients with larger levels of hematoma volume are at higher risk for neurological deterioration due to hematoma related complications [20, 23, 37]. Furthermore, it is well known that therapy of those patients requires more and prolonged intensive medical care and sometimes hematoma evacuation [10, 30, 35]. Accordingly, the impact of the clinical course after admission on the outcome is higher and the explanatory power of the admission NIHSS is lower in these patients. Further research is required to differentiate and exactly quantify the post-admission impact on functional outcome of ICH patients.

Furthermore, lack of assessment or underrepresentation of admission factors associated with functional outcome by the NIHSS might contribute to the mismatch between admission NIHSS and functional outcome. A subgroup analysis revealed higher ICH volume and brainstem or intraventricular location of ICH as independent factors associated with neurological deterioration, while younger age, normotension, lobar location, and lower volume of ICH were predictors for unexpected neurological improvement. All these factors are not fully apparent in NIHSS [1]. In addition, a ceiling effect is reported for NIHSS and may contribute to the particularly low explanatory power of the NIHSS in patients with larger ICH volume levels. According to this, the extent of neurological deficits in severely affected patients is falsely underreported by the NIHSS, since items that require patient’s cooperation cannot be tested [28].

In consequence, NIHSS on admission is a valid, but limited surrogate for evaluating the effect of hemorrhage volume on functional outcome in patients with smaller hematoma volumes. In patients with larger hemorrhage volumes, prognostic value of NIHSS at admission is further reduced since their outcome is more determined by factors not apparent in NIHSS on admission like secondary brain injury, adverse events, and treatment strategies. As previous studies have reported in relation to the ICH score, neurological status assessed by NIHSS at 24 or 48 h post-admission might be superior in prediction of functional outcome by taking clinical shifts that occurred up to that time, such as early neurological deterioration due to perihematomal edema, into account [2].

With regard to the unsatisfactory results of hemorrhage volume-reducing therapies [12, 26, 27], our results encourage the hypothesis of a cascade-like course of brain injury in ICH patients [19]. As in a domino effect, once the cascade has been started by the hematoma, it cannot be controlled anymore by volume reduction. Instead, our results encourage to focus on prevention of secondary deterioration, which have already led to a reduction in mortality in recent years [7, 17]. Interesting, upcoming concepts here are the utilization of coagulation factors to stop hematoma expansion (currently investigated, e.g., in the enrolling FASTEST trial, NCT03496883) or immunomodulators to target the inflammation [9].

## Limitations

Our study has several limitations. First, the data were retrospectively collected and were not part of a prospective clinical trial. Second, all clinical parameters including mRS and NIHSS were site-reported parameters that might suffer from site related bias due to limited interrater and intrarater reliability. Third, mRS assessment is heavily weighted toward motor functions, which might complicate comparisons of mRS and NIHSS. Fourth, only cases with availability of all required data points were included in the analysis. Exclusion of patients with missing data points might introduce bias to the reported results and might reduce generalizability of findings. Fifth, to generate valid results from mediation analysis, unmeasured confounding must not exist between parameters in the hypothetical causal model. This is a strong assumption, especially when considering that the many interconnected biological processes are not fully understood and might vary on patient-specific level. To minimize the effect of unmeasured confounding, we build the underlying regression models of the mediation analysis considering the known timely precedence and pathophysiological relations of the included variables. However, confounding cannot be precluded due to the observed unknown factors impacting functional outcome after successful recanalization. Sixth, the analysis does not include information regarding rehabilitation and other post-hospitalization factors. Consequently, the mediation model cannot differentiate the exact impact of other pre- and post-treatment factors that are not apparent in the neurological status at admission, including rehabilitation and late adverse events. Seventh, timing of discharge is not standardized and varies significantly between patients.

## Conclusion

In this multicenter analysis with 338 acute primary ICH patients, neurological deficits captured by NIHSS at admission reflect only 14% of the functional outcome at discharge for larger hematoma volumes and 30% of the functional outcome for smaller hematoma volumes. The majority of the clinical shifts that are significant for the outcome are not reflected in the NIHSS at admission. For clinical practice, these results indicate: (1) admission NIHSS has a very limited predictive value for the functional outcome of ICH patients; (2) the post-acute phase significantly contributes to functional outcome, underscoring the importance of the prevention of secondary brain injury and promotion of early rehabilitation.

## Abbreviations

aOR: Adjusted odds ratio
CI: Confidence interval
CTA: Computed tomography angiography
ICH: Intracerebral hemorrhage
IQR: Interquartile range
mRS: Modified Rankin Score
NECT: Non-enhanced computed tomography
NIHSS: National Institutes of Health Stroke Scale
ROI: Region of interest
SD: Standard deviation
